# Predictive model based on blood cell analysis and coagulation function indicators for neuroblastic tumors staging diagnosis

**DOI:** 10.3389/fonc.2025.1575863

**Published:** 2025-07-25

**Authors:** Yanzi Zhang, Lihong Zhang, Mengmeng Chen, Qin Dong, Chong Hu, Juan Wang, Jiao Meng, Xin Lv

**Affiliations:** ^1^ Clinical Laboratory, Children’s Hospital Affiliated to Shandong University, Jinan, China; ^2^ Clinical Laboratory, Jinan Children’s Hospital, Jinan, China; ^3^ Pathology Laboratory, Children’s Hospital Affiliated to Shandong University, Jinan, China; ^4^ Pathology Laboratory, Jinan Children’s Hospital, Jinan, China

**Keywords:** neuroblastoma, blood cell analysis, coagulation function indicators, staging diagnosis, predictive model

## Abstract

**Objective:**

To explore the diagnostic value of integrating blood cell analysis and coagulation function indicators in the staging of neuroblastic tumors, providing a robust basis for clinical decision-making.

**Methods:**

A retrospective analysis was conducted on 137 pediatric neuroblastic tumors cases (2017-2024) at the Children’s Hospital Affiliated to Shandong University. Patients were stratified into localized (INSS 1-2, Group 1) and advanced (INSS 3-4, Group 2) stages according to the INSS classification, with mature ganglioneuroma serving as the control group. Univariate and multivariate logistic regression analyses were performed to identify differences in blood cell analysis and coagulation function indicators between groups, complemented by ROC curve analysis to evaluate the efficacy of the models.

**Results:**

The median age of patients with neuroblastic tumor was 23.5 (12–46.75) months (male:female = 1.55:1), which was significantly younger than that of ganglioneuroma patients [72 (53–108) months, *p* < 0.01]. Multinomial logistic regression identified age, RDW-CV, Fib, and Hb as independent predictors of advanced stages. Older age, higher RDW-CV and Fib levels were positively associated with advanced-stage risk compare to localized stages, while higher Hb showed a negative association. Furthermore, a probability prediction model developed using age, TT, Mon#, and Hb successfully differentiated advanced neuroblastic tumors from ganglioneuroma. The overall accuracy of this prediction model was 78.10%, with specific accuracies of 68.40%, 82.40%, and 80.00% for the localized neuroblastic tumors, advanced neuroblastic tumors, and ganglioneuroma groups, respectively. ROC curves showed AUCs of 0.867 (localized vs. advanced) and 0.941 (advanced vs. ganglioneuroma), indicating high diagnostic efficacy.

**Conclusion:**

The combined analysis of age, RDW-CV, Hb, Mon#, Fib, and TT can effectively assist in the preliminary assessment of whether children with neuroblastic tumors are in an advanced phase or suffering from ganglioneuroma. This method enhances the accuracy and efficiency of clinical diagnosis and serves as a crucial reference for developing disease diagnosis and treatment plans.

## 1 Introduction

Neuroblastic tumor represents the most prevalent extracranial solid malignant tumor in children, constituting approximately 8%-10% of all childhood cancers. This tumor primarily affects children aged 0–4 years and typically originates from the adrenal medulla, abdominal ganglia, and sympathetic chain ganglia, among other locations (1–3). The significant heterogeneity of neuroblastic tumor, marked by variations in clinical presentation, histological morphology, genetic phenotypes, and clinical prognosis, complicates early diagnosis, the formulation of treatment strategies, and the standardization of protocols. Consequently, some children may present with malignant metastases at the time of diagnosis (4, 5). This not only complicates treatment but also results in a poor prognosis, severely jeopardizing the safety and quality of life of the affected children (6, 7). Therefore, early diagnosis and accurate staging of neuroblastic tumor are crucial for developing personalized treatment plans, evaluating prognosis, and enhancing patient survival rates.

With the continuous advancement of medical research, hematological parameters—such as blood cell analysis and coagulation function indicators—have garnered significant attention for their potential value in tumor diagnosis and staging. As routine clinical tests, these indicators offer several advantages, including convenient detection, low cost, and high repeatability. Numerous studies have demonstrated that the occurrence and progression of tumors are closely linked to abnormal changes in both the coagulation and hematopoietic systems. For instance, in tumors such as ovarian cancer and hepatocellular carcinoma, thrombin time (TT) has been implicated in the microvascular invasion and metastasis of tumor cells (8, 9), while fibrinogen (Fib) can enhance the metastatic potential of tumor cells by inhibiting natural killer (NK) cell-mediated clearance of tumor cells within blood vessels (10). However, the combined predictive role of blood cell analysis and coagulation function indicators in the staging and tissue typing of neuroblastic tumor has not been thoroughly investigated. The objective of this study is to conduct an in-depth analysis of the association between blood cell analysis and coagulation function indicators and the different stages and tissue types of neuroblastic tumors, with the hope of discovering a combination of biomarkers with clinical value, thereby improving the accuracy and reliability of neuroblastic tumors staging and tissue typing. This study aims to establish a more robust theoretical foundation for clinicians to develop personalized treatment plans and implement early interventions, thereby enhancing the prognosis of children with neuroblastic tumor.

## 2 Materials and methods

### 2.1 Case data

This study collected the basic clinical data of 183 children who were first diagnosed with neuroblastic tumors at our hospital between April 1, 2017, and April 30, 2024. To ensure the scientificity and accuracy of the research, we established stringent inclusion and exclusion criteria.

The inclusion criteria for this study are as follows: (1) Tumor tissue must be confirmed through optical microscopy (11); (2) A bone marrow biopsy or aspirate must reveal characteristic neuroblastic tumor cells, which are small round cells organized in nests or chrysanthemum-like clusters, or exhibit positive staining for the anti-GD2 antibody, accompanied by elevated levels of urinary vanillylmandelic acid (VMA) and serum neuron-specific enolase (NSE) (11); (3) Complete clinical data must be available for all participating children; (4) Children must not have received any treatment prior to admission. Enrolled pediatric patients are required to meet the following mandatory criteria: they must fulfill at least one of the requirements outlined in either criterion (1) or (2) and simultaneously satisfy both criteria (3) and (4).

The exclusion criteria for this study are as follows: (1) Children who had received radiotherapy, chemotherapy, immunotherapy, endocrine therapy, anti-infective therapy, or any other treatments prior to admission; (2) Children with acute or chronic diseases affecting the liver, kidneys, or blood system, or other conditions that could influence coagulation and blood cell analysis indicators; (3) Children with a history of blood transfusion within 8 weeks before admission; (4) Children with incomplete clinical and pathological data. Any children meeting one or more of the above criteria were excluded from the study.

Based on these criteria, a total of 137 children with neuroblastic tumors were included in this study, classified according to the International Neuroblastoma Pathology Classification (INPC) (12). This cohort included 88 cases of neuroblastoma, 24 cases of ganglioneuroblastoma, and 25 cases of ganglioneuroma. According to the staging criteria of the International Neuroblastoma Staging System (INSS) (11), there were 20 cases at stage 1, 18 cases at stage 2, 30 cases at stage 3, and 44 cases at stage 4, along with the aforementioned 25 cases of ganglioneuroma. In this study, patients with neuroblastic tumors, encompassing both neuroblastoma and ganglioneuroblastoma, were classified into two groups according to INSS: Group 1, which includes patients at INSS stages 1-2 (the localized stages group), and Group 2, which comprises patients at INSS stages 3-4 (the advanced stages group). Furthermore, a control group consisting of mature-type ganglioneuroma was included for comparative analysis. The clinical data collected from the children included gender, age at initial diagnosis, residential area, tissue type, stage of disease, and hematological indicators to be observed (the indicators included in the observation were the blood cell analysis and coagulation function indicators at the time of initial diagnosis and prior to treatment).

**Figure 1:**
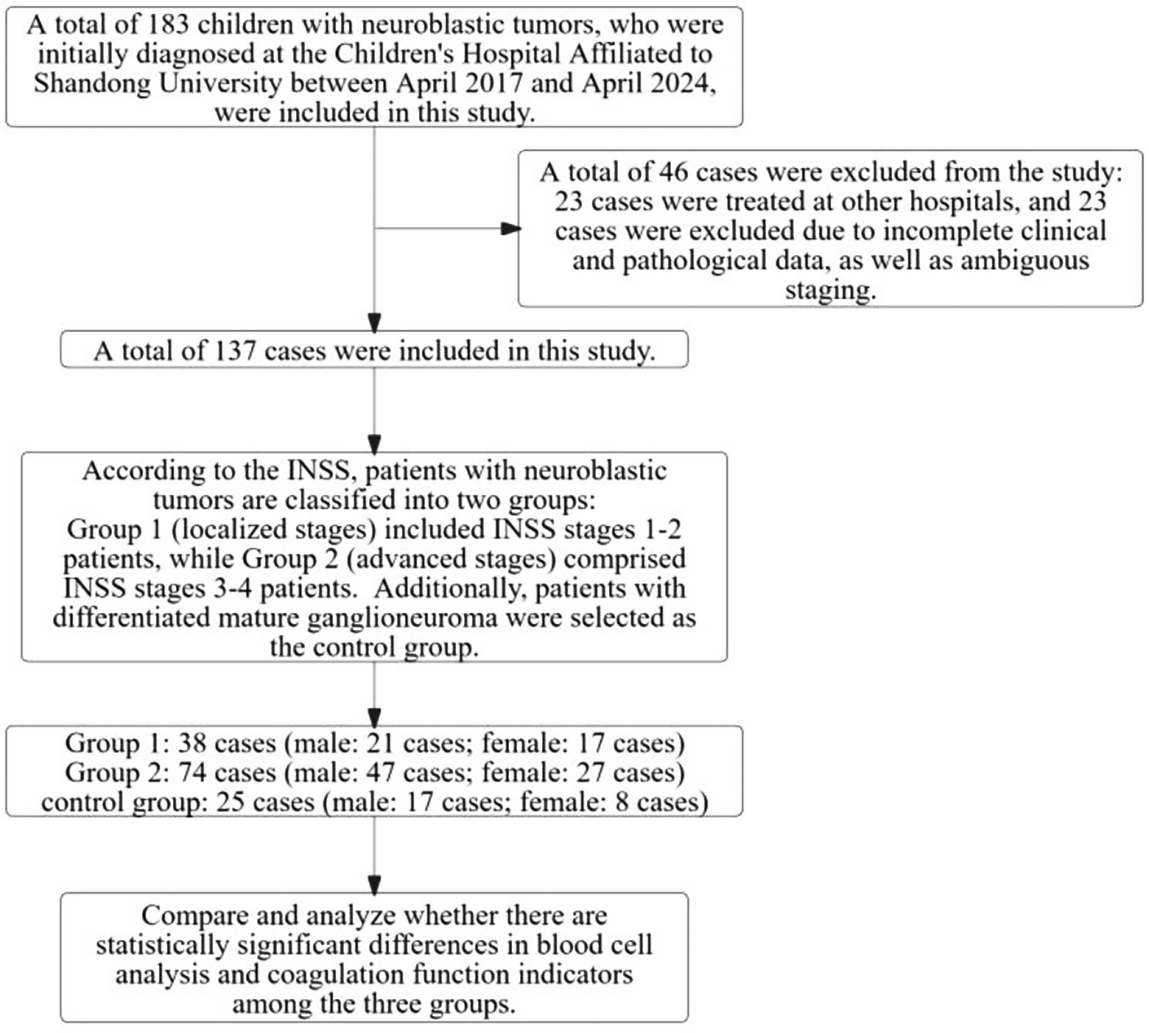
Study process for neuroblastic tumors in children: case inclusion, exclusion, and grouping.

This study has obtained ethical approval from the Ethics Committee of the Children’s Hospital Affiliated to Shandong University. Since all the tests were part of routine diagnosis and treatment, participants were not required to provide informed consent. Laboratory data were anonymized prior to analysis. The specific details of enrollment and grouping are presented in **Figure 1**.

### 2.2 Experimental methods

In this retrospective study, we collected and analyzed the results of blood cell analysis and coagulation function indicators from the child’s initial visit (for detailed indicators, please refer to **Table 1**). Blood cell analysis was conducted using the Sysmex XN1000 system, while the Sysmex CS5100 system was employed to evaluate the child’s coagulation function indicators. To ensure the accuracy and reliability of the test results, the staff meticulously checked the operating conditions of the instruments prior to each test, confirming that they were within acceptable ranges and that the test reagents were normal and usable.

**Table 1:** Investigating differences in basic information and laboratory indicators among children in three research groups: a comparative analysis.

Indicators	Group 1 (n=38)	Group 2 (n=74)	Ganglioneuroma (n=25)	F/H/x^2^	*p*
age (months)	12 (2.75∼35.25)	35.0 (12.0∼48.0)^a^	72.0 (53.0∼108.0)^bc^	50.771	**<0.001**
sex				1.186	0.553
male	21 (55.26%)	47 (63.51%)	17 (68.0%)		
female	17 (44.74%)	27 (36.49%)	8 (32.0%)		
Regions				1.156	0.469
rural	22 (57.89%)	35 (47.3%)	11 (44.0%)		
urban	16 (42.11%)	39 (52.1%)	14 (56.0%)		
TT (s)	18.5 (17.38∼20.18)	17.9 (16.38∼19.6)	17.1 (16.05∼18.3)^b^	6.193	**0.045**
Fib (g/L)	2.1 (1.78∼2.64)	2.58 (1.97∼4.24)^a^	2.3 (1.97∼2.79)	8.781	**0.012**
PT (s)	12 (11.58∼12.73)	12.8 (12.08∼13.7)^a^	12.1 (11.7∼12.75)	13.392	**0.001**
INR (%)	1.02 (0.97∼1.07)	1.07 (1.02∼1.16)^a^	1.03 (1∼1.08)	12.06	**0.002**
D-Dimer (g/L)	0.42 (0.26∼1.13)	2.49 (0.64∼8.3)^a^	0.21 (0.14∼0.33)^bc^	41.181	**<0.001**
WBC (×10^9^/L)	8.63 (6.89∼10.74)	8.26 (6.46∼10.46)	6.94 (6.09∼8.81)	5.687	0.058
LYM# (×10^9^/L)	4.76 (3.46∼6.75)	3.4 (2.17∼5.04)^a^	2.93 (2.5∼3.98)^b^	18.269	**<0.001**
NEU# (×10^9^/L)	2.34 (1.73∼3.66)	3.43 (2.41∼5.57)^a^	3.16 (2.28∼4.34)	8.154	**0.017**
MON# (×10^7^/L)	51.5 (45.0∼70.25)	55.0 (38.75∼81.5)	37.0 (34.0∼49.5)^bc^	14.701	**0.001**
BASO# (×10^9^/L)	0.03 (0.02∼0.04)	0.02 (0.02∼0.04)	0.03 (0.02∼0.04)	1.798	0.407
EOS# (×10^9^/L)	0.28 (0.08∼0.4)	0.12 (0.04∼0.2)^a^	0.13 (0.08∼0.22)	15.215	**<0.001**
RBC (×10^12^/L)	4.34 (4.03∼4.63)	4.06 (3.52∼4.39)	4.59 (4.31∼4.71)^c^	17.579	**<0.001**
Hb (g/L)	119.58 ± 14.11	101.59 ± 21.4^a^	124.88 ± 11.23^c^	21.645	**<0.001**
MCV (fl)	82.5 (79.83∼86.93)	79 (75.95∼84.55)^a^	82.7 (80.5∼84.5)	11.967	**0.003**
RDW-CV (%)	13.2 (12.3∼14.3)	14.1 (12.9∼15.45)^a^	12.3 (11.9∼13.15)^c^	26.199	**<0.001**
PLT (×10^9^/L)	364.34 ± 107.4	348.82 ± 134.64	329.36 ± 100.24	0.624	0.537
MPV (fl)	9.33 ± 1.03	9.05 ± 1.05	9.3 ± 0.86	1.206	0.303
PDW (%)	12.75 (9.48∼15.9)	12 (9.4∼15.6)	11.1 (9.45∼15.65)	0.9	0.638
PLCR (%)	18.9 (15.15∼25.93)	17.2 (14∼22.83)	19.5 (15.2∼25.4)	3.666	0.16
NLR	0.47 (0.35∼0.75)	0.96 (0.59∼2.21)^a^	1.15 (0.64∼1.42)^b^	21.713	**<0.001**
EMR	0.43 (0.15∼0.76)	0.17 (0.07∼0.4)^a^	0.44 (0.17∼0.57)^c^	14.812	**0.001**
LMR	9.09 (6.18∼11.51)	6.77 (3.97∼9.38)^a^	7.89 (6.36∼9.94)	8.051	**0.018**

TT, Thrombin Time; Fib, Fibrinogen; PT, Prothrombin Time; INR, International Normalized Ratio; WBC, White Blood Cell; LYM#, Lymphocyte Count; NEU#, Neutrophil Count; MON#, Monocyte Count; BASO#, Basophil Count; EOS#, Eosinophil Count; RBC, Red Blood Cell; Hb, Hemoglobin; MCV, Mean Corpuscular Volume; RDW-CV, Red Cell Distribution Width-Coefficient of Variation; PLT, Platelet; MPV, Mean Platelet Volume; PDW, Platelet Distribution Width; PLCR, Platelet-large Cell Ratio; NLR=NEU#/LYM#; MER=EOS#/MON#; LMR= LYM#/MON#.

^a^ *p <*0.05 compared to Group 1; ^b^ *p <*0.05 compared to Group 1; ^c^ *p <*0.05 compared to Group 2. The bold p-values in the table indicate statistically significant differences in the comparisons between groups (p < 0.05).

### 2.3 Statistical analysis

Statistical analysis of the data was performed using SPSS 25.0. Count data are expressed as rates (%), and measurement data that conforming to a normal distribution are expressed as $\left(\bar{x}\pm SD\right)$ , while measurement data not conforming to a normal distribution are expressed as Q50 (Q25~Q75). In univariate analysis, count data were analyzed using the X^2^ test, measurement data following a normal distribution were analyzed using one-way ANOVA, and measurement data not following a normal distribution were analyzed using the Kruskal-Wallis H test for statistical difference analysis. For multivariate analysis, an unordered multinomial logistic regression analysis was employed using a backward stepwise approach to construct a probabilistic predictive model. Receiver Operating Characteristic (ROC) curves were plotted to assess the diagnostic efficacy of both the model and each individual indicator. In this study, a statistical difference was considered significant when *p* < 0.05.

## 3 Experimental results

### 3.1 Clinical manifestations of children at initial presentation

**Figure 2:**
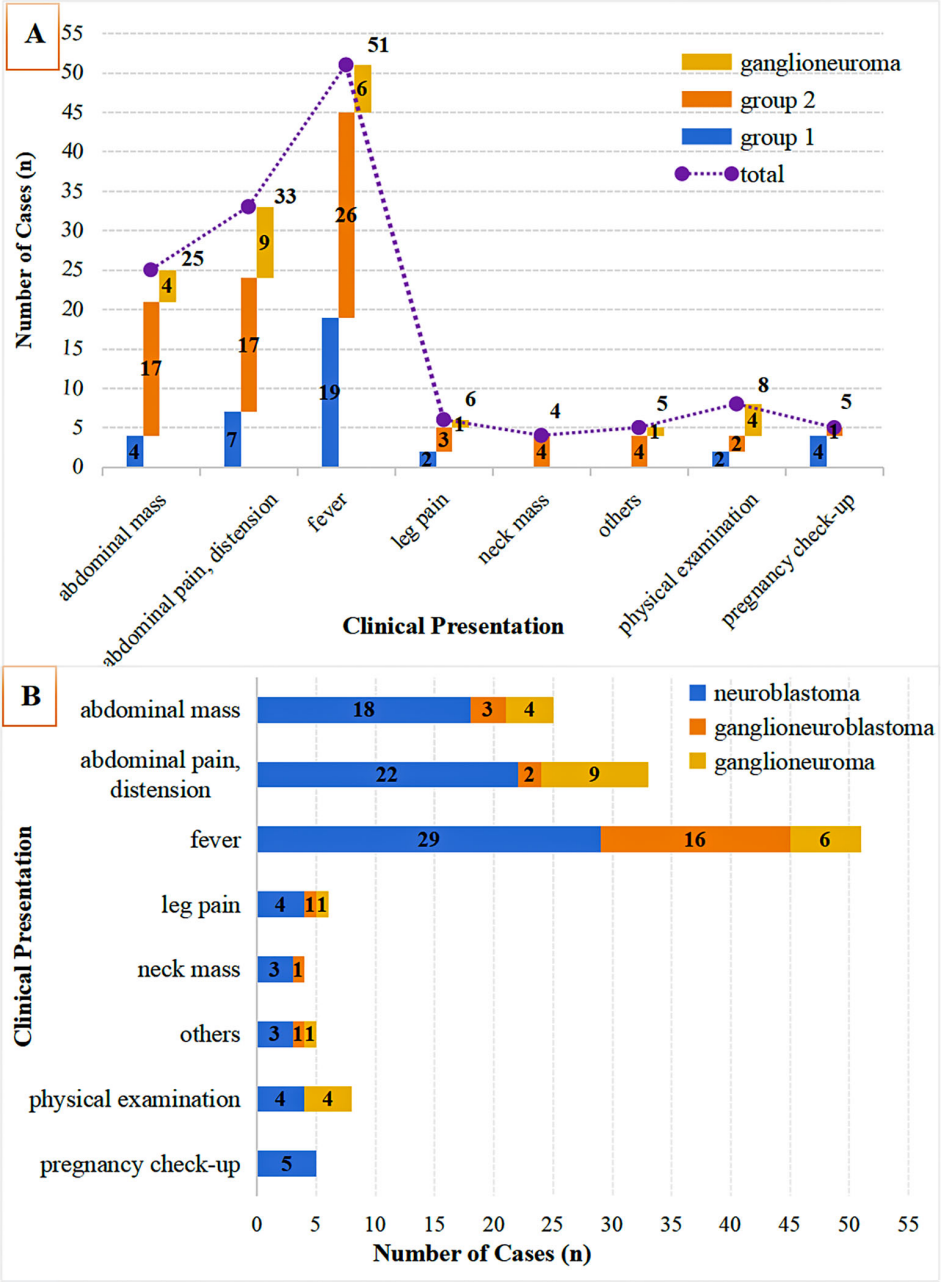
Clinical symptom distribution by tumor stage & histology. **(A)** Initial symptoms in neuroblastic tumors (Group 1: INSS 1-2; Group 2: INSS 3-4) vs. ganglioneuroma; **(B)** Symptom distribution across histologies (neuroblastoma, ganglioneuroblastoma, ganglioneuroma).

As illustrated in **Figure 1**, this study initially enrolled 183 children diagnosed with neuroblastic tumors at the Children’s Hospital Affiliated to Shandong University between 2017 and 2024. After applying the established exclusion criteria, 46 cases were excluded, resulting in a final cohort of 137 children for analysis. According to the INSS, the selected children were categorized into localized-stage (n=38 cases, INSS stages 1-2) and advanced-stage (n=74 cases, INSS stages 3-4). Furthermore, a control group of 25 children diagnosed with ganglioneuroma was included. As shown in **Figure 2**, the clinical symptoms of neuroblastic tumor are significantly associated with tumor stage and histological type. **Figure 2A** illustrates that advanced-stage tumors (Group 2, INSS stages 3-4) are more likely to present with symptoms related to local invasion and metastasis. The incidence of abdominal mass in advanced-stage tumors is significantly higher (23.0%, 17/74) compared to localized-stage tumors (Group 1, 10.5%) and ganglioneuroma (GN, 16.0%). Additionally, manifestations of metastasis, such as leg pain and neck mass, were observed. Fever is the most common symptom, reported in 51 cases, with a higher incidence in localized-stage children (50.0%, 19/38) compared to advanced-stage (35.1%, 26/74) and GN (24.0%, 6/25). This difference may be related to the inflammatory response triggered by early-stage tumors. From the perspective of tissue types (**Figure 2B**), GN is predominantly characterized by abdominal pain or distension (36.0%, 9/25) and incidental findings during physical examinations (16.0%, 4/25), indicating its indolent biological behavior. In contrast, neuroblastoma (NB) is more frequently associated with metastatic symptoms (leg pain 4.55%, neck mass 3.41%), while ganglioneuroblastoma (GNB) has the highest incidence of fever (66.67%, 16/24), reflecting the heterogeneity between tumor differentiation and clinical manifestations.

### 3.2 Single-factor analysis of laboratory parameters in different groups

**Figure 3:**
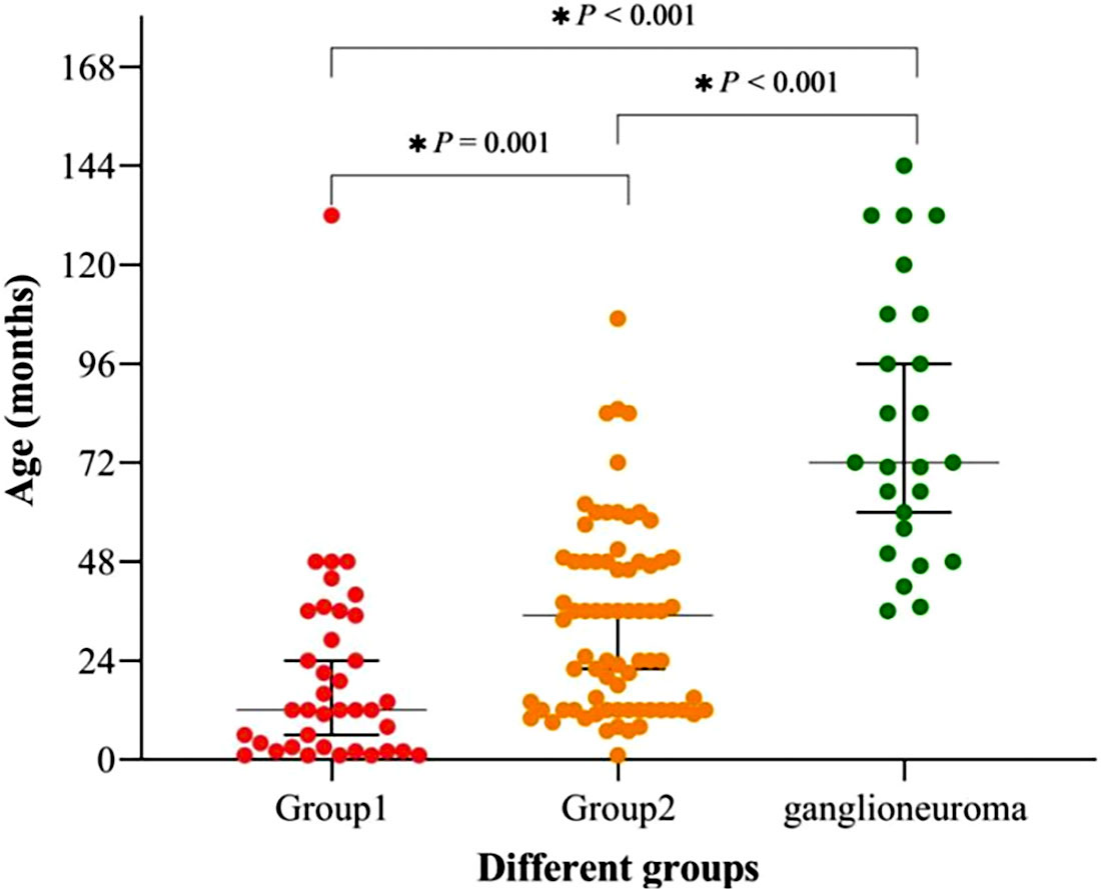
Age distribution and intergroup comparisons in Group 1, Group 2, and Ganglioneuroma. Statistically significant differences (*p* < 0.05) were observed: Group 1 vs. Ganglioneuroma: *p* < 0.001; Group 1 vs. Group 2: *p* = 0.001; Group 2 vs. Ganglioneuroma: *p* < 0.001).

This study involved a total of 112 children diagnosed with neuroblastic tumors, comprising 68 males and 44 females, resulting in a male-to-female ratio of 1.55:1. As illustrated in **Figure 1**, patients with neuroblastic tumors (including neuroblastoma and ganglioneuroblastoma) were stratified into Group 1 (localized stages, INSS stages 1-2) and Group 2 (advanced stages, INSS stages 3-4) according to the INSS. Notably, no significant difference was observed in the distribution of neuroblastic tumor histological subtypes between groups (Chi-square test, *p* > 0.05). In this study, the age of onset for ganglioneuroma was found to be significantly later than that for neuroblastic tumors, with median ages of 72 (53-108) months and 23.5 (12-46.75) months for ganglioneuroma and neuroblastic tumors, respectively (**Supplementary Table 1**). As depicted in **Table 1** and **Figure 3**, the age distribution followed the order: ganglioneuroma > Group 2 > Group 1. Concurrently, D-Dimer values exhibited a gradient of ganglioneuroma < Group 1 < Group 2, reflecting a progressive increase with tumor advancement. Furthermore, the values of Fib, prothrombin time (PT), international normalized ratio (INR), neutrophil count (NEU#), red cell distribution width-coefficient of variation (RDW-CV), and neutrophil-to-lymphocyte ratio (NLR) exhibited a gradually increasing trend with tumor progression, whereas the values of lymphocyte count (Lym#), eosinophil count (EOS#), hemoglobin (Hb), mean corpuscular volume (MCV), eosinophil-to-monocyte Ratio (EMR), and lymphocyte-to-monocyte ratio (LMR) demonstrated a gradual decrease with tumor progression. Compared to Group 1, the values of TT, lymphocyte count (LYM#), and monocyte count (MON#) in the GN group were lower, while the NLR was higher. In comparison to Group 2, the ganglioneuroma group exhibited higher red blood cell count (RBC), Hb, and EMR, but lower MON# and RDW-CV. No statistically significant differences were found in the other observed indicators among the three groups.

### 3.3 Unordered multinomial logistic regression analysis (backward stepwise) for different groups

In this section, we employed unordered multinomial logistic regression with backward stepwise variable selection to analyze the observational indicators that demonstrated statistical significance in the univariate analysis (see **Table 1**). These indicators exhibited a variance inflation factor (VIF) of less than 5, and detailed results of the collinearity diagnosis are available in **Supplementary Table 2**. Group 2 was designated as the reference group. The analysis aimed to identify associated factors between this group and the ganglioneuroma group, specifically Group 1. The objective was to examine the presence of independent influencing indicators between each pair of groups.

Through unordered multinomial logistic regression analysis comparing Group 1 and Group 2, age (months), Fib, RDW-CV, and Hb were identified as independent predictors of neuroblastic tumor progression to the advanced stage (VIF < 5; for collinearity diagnostics of the indicators, refer to **Supplementary Table 3**). The regression coefficients (B values) for age (months), Fib, and RDW-CV were -0.049, -0.774, and -0.493, respectively, all of which are less than 0, with *p* < 0.05, corresponding to OR <1 (age (months): OR = 0.953, 95% CI: 0.924-0.982; Fib: OR = 0.461, 95% CI: 0.219-0.970; RDW-CV: OR = 0.611, 95% CI: 0.391-0.995). This indicates that higher values of age (months), Fib, and RDW-CV are associated with a reduced likelihood of being classified into Group 1 (i.e., a higher probability of belonging to Group 2). In contrast, the regression coefficient (B value) for Hb was 0.044 (OR = 1.045, 95% CI: 1.006-1.085, *p* < 0.05). A positive B value corresponds to OR > 1, meaning increased Hb levels are associated with a higher likelihood of being classified into Group 1 (i.e., a lower probability of belonging to Group 2) (see **Table 2** for detailed statistics). In the comparison between the GN group and Group 2, age (months), Hb, TT, and Mon# (×10^7^/L) were identified as independent predictors of the grouping (VIF < 5; for collinearity diagnostics of the indicators, refer to **Supplementary Table 3**). The regression coefficients (B values) for age (months) and Hb were 0.022 (OR = 1.022, 95% CI: 1.002-1.043) and 0.07 (OR = 1.073, 95% CI: 1.010-1.138). Positive B values imply OR > 1, indicating that higher values of age (in months) and Hb levels are associated with an increased likelihood of being classified into the GN group (vs. Group 2). Conversely, the regression coefficients (B values) for TT and Mon# (×10^7^/L) were -0.611 (OR = 0.543, 95% CI: 0.331-0.890) and -0.064 (Mon#: OR = 0.938, 95% CI: 0.889-0.990), respectively. The negative B values indicate that OR < 1, suggesting that higher levels of TT and Mon# are associated with a reduced likelihood of being classified into the GN group, which in turn implies a higher probability of belonging to Group 2 (detailed results are shown in **Table 2**). Furthermore, the p-values for the other observations were all greater than 0.05, indicating a lack of statistical significance.

**Table 2:** Unordered multi-category logistic regression analysis results of three groups of data.

Group	Variable	B	Std. Error	Wald	*p*	OR	95% CI	
group 1* (n=38)	intercept	9.725	5.502	3.125	0.077			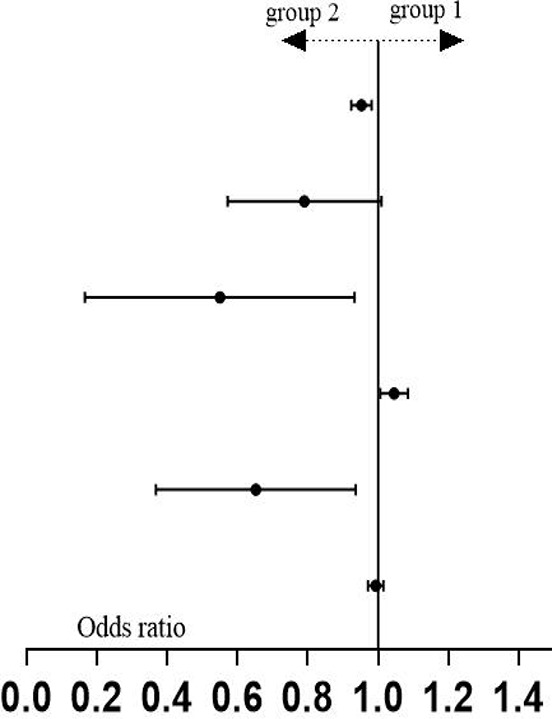
	age (months)	-0.049	0.016	9.814	**0.002**	0.953	0.924-0.982	
	TT	-0.26	0.142	3.346	0.067	0.771	0.583-1.019	
	Fib	-0.774	0.379	4.168	**0.041**	0.461	0.219-0.970	
	Hb	0.044	0.019	5.142	**0.023**	1.045	1.006-1.085	
	RDW-CV	-0.493	0.228	4.675	**0.031**	0.611	0.391-0.955	
	Mon# (×10^7^/L)	-0.006	0.011	0.328	0.567	0.994	0.972-1.016	
ganglion-euroma* (n=25)	intercept	4.967	8.158	0.371	0.543			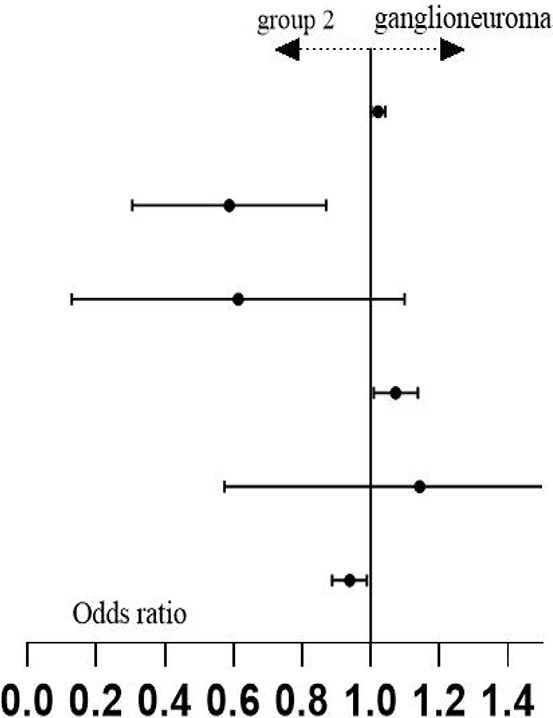
	age (months)	0.022	0.01	4.629	**0.031**	1.022	1.002-1.043	
	TT	-0.611	0.252	5.863	**0.015**	0.543	0.331-0.890	
	Fib	-0.72	0.439	2.69	0.101	0.487	0.206-1.151	
	Hb	0.07	0.03	5.295	**0.021**	1.073	1.010-1.138	
	RDW-CV	0.047	0.262	0.033	0.856	1.049	0.627-1.754	
	Mon# (×10^7^/L)	-0.064	0.027	5.474	**0.019**	0.938	0.889-0.990	

*The reference category is: Group 2; Pseudo R -square: Cox and Snel = 0.560, Nagelkerke = 0.648, McFadden = 0.411. The bold p-values in the table indicate that the results of the regression analysis for this indicator are statistically significant (p < 0.05), suggesting that this indicator functions as an independent predictor of intergroup differences. $\frac{Pgroup1}{Pgroup2}=e^{\text{(9.725-0.049*age(months)-0.26*TT-0.774*Fib+0.044*Hb-0.493*RDW-CV-0.006*Mon\#(*10\textasciicircum{}7/L)}}$ ; $\frac{P\text{ganglioneuroma}}{Pgroup2}=e^{\left(4.967+0\text{.022*age(months)-0}.611*TT-0.72*Fib+0.07*Hb+0.047*RDW-CV-0\text{.064*Mon\#(*10\textasciicircum{}7/L}\right)}$ .

### 3.4 Classification accuracy of probabilistic models and analysis of ROC curve

**Figure 4:**
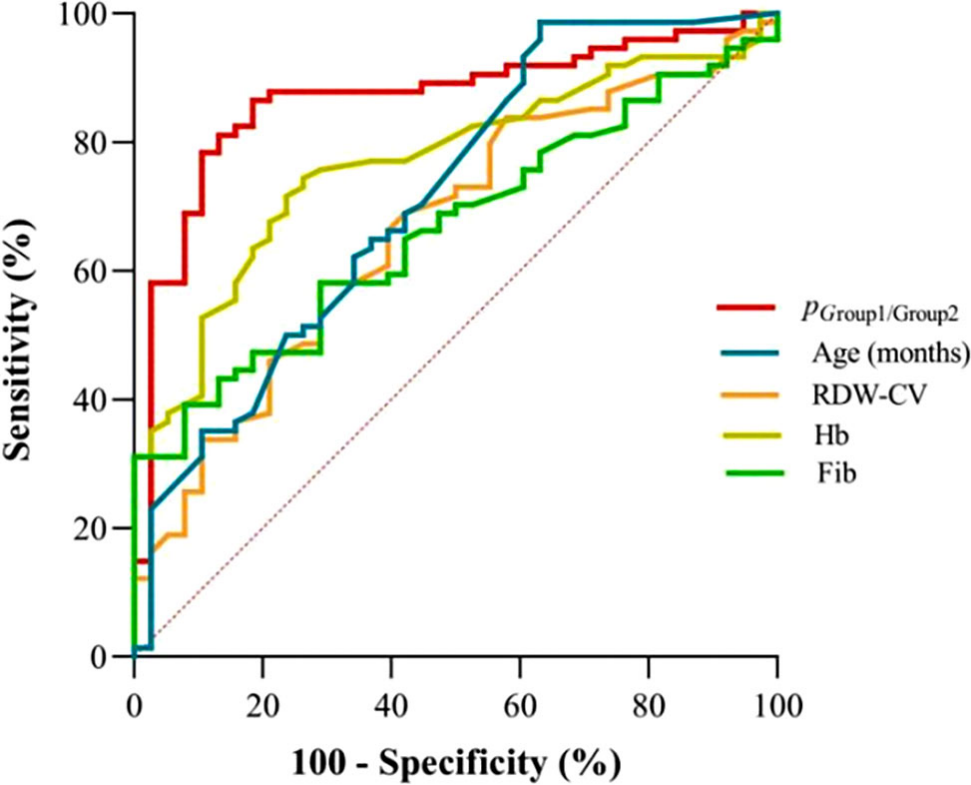
ROC curves for indicators in discriminating localized (Group 1) and advanced-stage neuroblastic tumors (Group 2). (Definition: *p*_(Group1/Group2)_ denotes the probability output by the multinomial logistic regression model for classifying cases as localized (Group 1) or advanced-stage (Group 2) neuroblastic tumors).

**Figure 5:**
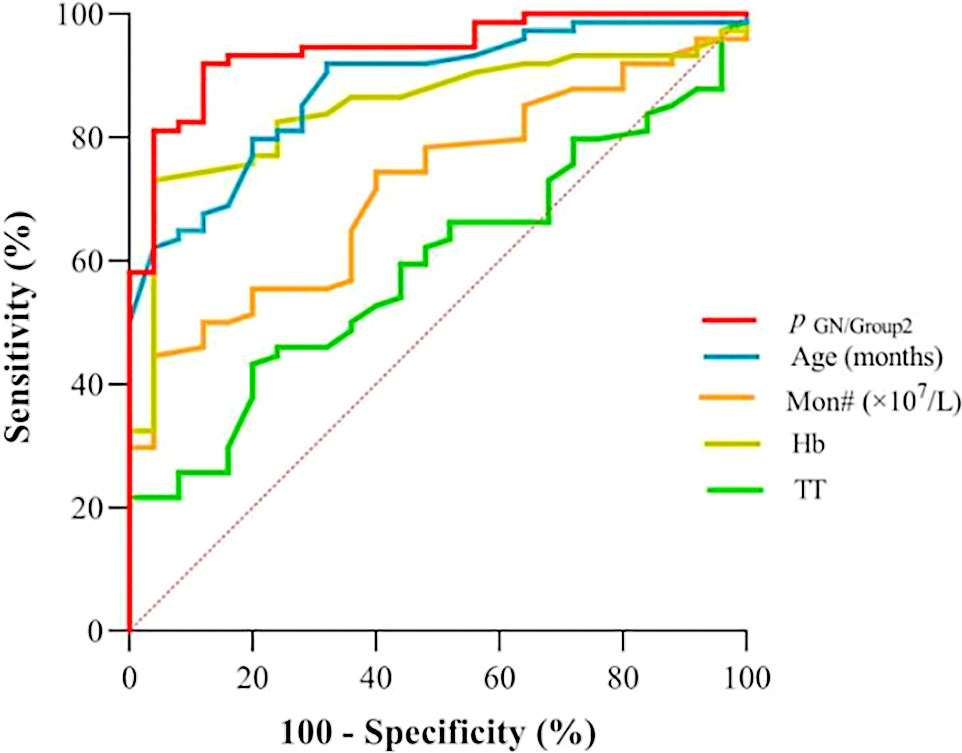
ROC curves for indicators in discriminating ganglioneuroma (GN) from advanced-stage neuroblastic tumors (Group 2). (GN: Ganglioneuroma; Definition: *p*_(GN/group2)_ denotes the probability output by the multinomial logistic regression model for classifying cases as GN or advanced-stage (Group 2) neuroblastic tumors).

According to **Table 3**, the probability prediction model developed in this study demonstrates an overall diagnostic accuracy of 78.1% (error rate: 21.9%) in differentiating between neuroblastic tumor stages and ganglioneuroma. Specifically, the model achieves classification accuracies of 68.4% for localized neuroblastic tumors (INSS 1–2, Group 1), 82.4% for advanced neuroblastic tumors (INSS 3–4, Group 2), and 80.0% for ganglioneuroma. The higher accuracy in Group 2 compared to Group 1 may reflect the more distinct biological characteristics of advanced tumors, while the moderate accuracy in Group 1 could be attributed to the heterogeneity of early-stage lesions. ROC curve analysis reveals the model’s superior discriminative ability. For distinguishing localized from advanced neuroblastic tumors, the area under the curve (AUC) is 0.867 (95% CI: 0.796–0.938), with a cutoff value of 0.879 yielding a sensitivity of 86.5% and specificity of 81.6% (**Figure 4**, **Table 4**). Notably, Hb (AUC=0.769) and RDW-CV (AUC=0.666) exhibit moderate single-marker efficacy, underscoring the added value of combined indicators. For differentiating advanced neuroblastic tumors from ganglioneuroma, the model achieves an AUC of 0.941 (95% CI: 0.894–0.988), with sensitivity and specificity of 91.9% and 88.0%, respectively (**Figure 5**, **Table 5**). Age (AUC=0.880) and Hb (AUC=0.849) are identified as key contributors, consistent with the distinct age distribution and hematological profiles between malignant and benign tumors. As illustrated in **Figures 4** and **5**, the model’s ROC curves substantially outperform individual markers, with 95% CIs indicating stable diagnostic efficacy. The optimal cutoff values balance sensitivity and specificity for clinical application, providing a robust tool for initial staging and histological differentiation in pediatric neuroblastic tumor patients.

**Table 3:** Assessment of the model’s accuracy in classifying observation samples.

Observed data	Predicted data				
	Group 1	Group 2	Ganglioneuroma	Accuracy Rate	Error Rate
Group 1	26	10	2	68.4%	31.6%
Group 2	8	61	5	82.4%	17.60%
ganglioneuroma	1	4	20	80.0%	20.00%
Overall percentage				78.1%	21.9%

1. The model was constructed based on unordered multinomial logistic regression, incorporating indicators such as age, TT, Fib, Hb, and RDW - CV as predictive variables.

2. n = 137, including Group 1 (38 cases), Group 2 (74 cases), and ganglioneuroma (25 cases).

**Table 4:** ROC Efficacy of indicators for localized vs advanced neuroblastic tumors (group1 vs group2).

Indicators	AUC	*p*	95% CI	CUT OFF	Sensitivity	Specificity
*P*_(Group1/Group2)_	0.867	< 0.001	0.796~0.938	0.879	86.5%	81.6%
age (months)	0.714	< 0.001	0.611~0.816	6.5	98.6%	36.8%
RDW-CV	0.666	0.004	0.562~0.769	13.75	58.1%	70.8%
Hb	0.769	< 0.001	0.681~0.856	114.5	74.3%	73.7%
Fib	0.666	0.004	0.567~0.764	3.035	39.2%	92.1%

**Table 5:** ROC efficacy of indicators for GN vs advanced neuroblastic tumors (GN vs group2).

Indicators	AUC	*p*	95% CI	CUT OFF	Sensitivity	Specificity
*p*_(GN/group2)_	0.941	< 0.001	0.894~0.988	0.544	91.9%	88.0%
age (months)	0.88	< 0.001	0.812~0.949	63.5	91.9%	68.0%
Mon# (×10^7^/L)	0.718	0.001	0.615~0.821	57.5	44.6%	96.0%
Hb	0.849	< 0.001	0.77~0.928	113.5	73.0%	96.0%
TT	0.59	0.18	0.473~0.707	18.45	43.2%	80.0%

These figures clearly indicate that the model exhibits a high level of accuracy in predicting the staging of newly diagnosed neuroblastic tumor patients. This accuracy provides a reliable foundation for precise initial staging and the formulation of subsequent treatment plans. Furthermore, by optimizing the timeliness and precision of staging diagnoses, the model assists clinicians in developing personalized treatment strategies for patients at earlier stages. This establishes a crucial basis for enhancing overall therapeutic outcomes and improving the quality of life for patients with neuroblastic tumors.

## 4 Discussion

Neuroblastic tumors exhibit significant clinical heterogeneity, often presenting with malignant metastasis at the time of diagnosis. This heterogeneity is underscored by stark prognostic disparities: extremely low-risk and low-risk patients (International Neuroblastoma Risk Group [INRG] (13)) achieve over 95% five-year survival with conservative therapy (1, 14, 15), while high-risk cases demonstrate less than 60% five-year survival despite intensive treatment (6, 7), underscoring the critical need for early staging. Clinically, this heterogeneity manifests in distinct symptomatic profiles: advanced-stage tumors (INSS 3-4) typically present as abdominal masses accompanied by metastatic symptoms (e.g., leg pain), whereas localized-stage tumors (INSS 1-2) are characterized by a higher incidence of fever. In contrast, benign ganglioneuroma primarily causes abdominal pain or is detected incidentally, reflecting its indolent biology (**Figure 2**). Our study contributes to this understanding by identifying age as a paradoxical predictor: neuroblastic tumors predominantly affect children aged 0–48 months (male:female ratio = 1.55:1), while ganglioneuroma presents at a median age of 72 months (*p* < 0.01). Older age is associated with both advanced-stage tumors (OR = 0.953) and ganglioneuroma (OR = 1.022), a paradox that can be explained by the fetal origin of neuroblastic tumors and the differentiated maturation of ganglioneuroma (12, 16, 17). Mechanistically, this age association may interact with hematological markers (e.g., RDW-CV, Hb) identified in our model. For instance, age-related changes in the marrow microenvironment may exacerbate RDW-CV elevation in advanced tumors (OR = 0.611), while declining sensitivity to erythropoietin could worsen Hb reduction (18, 19). Such interactions support the model’s efficacy (AUC = 0.867) and advocate for the integration of age and hematological factors in non-invasive staging. This integrative approach is further validated by the distinct roles of additional hematological and coagulation markers, which collectively enhance the discriminatory power between tumor stages and histotypes.

Furthermore, our research demonstrates that the combined assessment of RDW-CV, TT, Mon#, Fib, and Hb holds significant clinical value in evaluating the disease staging of newly diagnosed neuroblastic tumors in children and differentiating them from ganglioneuroma. In recent years, numerous studies have demonstrated that blood cell analysis and coagulation function indicators are increasingly significant in tumor staging and prognosis. For instance, across diverse cancer types-including hepatocellular carcinoma (20), pancreatic ductal adenocarcinoma (PDAC) (21, 22), head and neck cancers (23), and prostate cancer (24)-elevated monocyte counts have been identified as independent predictors of postoperative recurrence and poor patient outcomes. This study extends these findings to neuroblastoma, demonstrating that monocyte count (Mon#) also serves as an independent factor differentiating ganglioneuroma from neuroblastic tumors at advanced stages. In particular, for every 1- unit increase in Mon# (×10^7^/L), the risk of patients being categorized into the ganglioneuroma group (versus Group 2 as the reference) decreased by 6.2%. Notably, existing research has indicated that untreated neuroblastic tumors tissues are enriched with tumor-associated macrophages (TAM), tumor-associated fibroblasts (CAF), and their precursor mesenchymal stromal cells (MSC), which interact with neuroblastic tumors cells to induce the production of cytokines such as transforming growth factor-β1 (TGF-β1), monocyte chemoattractant protein-1 (MCP-1), interleukin-6 (IL-6), and interleukin-8 (IL-8) (25–27). These interactions suppress the activity of immune cells, including T lymphocytes and natural killer (NK) cells, while simultaneously activating the TGF-β1/IL-6 signaling pathway, which prevents the spontaneous apoptosis of monocytes (MN). Furthermore, in response to neuroblastic tumors cells, monocytes can differentiate into TAM, which can promote tumor cell invasion through a paracrine mechanism involving colony-stimulating factor 1 (CSF-1) and epidermal growth factor (EGF) (25–27). Additionally, TAMs secrete vascular endothelial growth factor (VEGF), which stimulates tumor angiogenesis to supply oxygen and nutrients, thereby accelerating tumor growth and metastatic dissemination (28). This study also demonstrates a significant association between elevated RDW-CV and neuroblastic tumors in advanced stages. Specifically, for each 1-unit increase in RDW-CV, the likelihood of neuroblastic tumor patients being classified with localized tumors rather than advanced tumors decrease by 38.9%. Several factors may contribute to this phenomenon: First, the bone marrow, bones, and liver are the most common sites of metastasis for neuroblastic tumors (29). When neuroblastic tumor cells metastasize to these sites, they can deprive the body of nutrients, disrupt red blood cell production, and consequently lead to an increase in RDW-CV. Second, neuroblastic tumor cells, along with inflammatory mediators such as IL-6, IL-8 and TGF-β1 (30) produced by bone marrow stromal cells (BMSC) stimulated by these cells, can interfere with the normal process of red blood cell production, leading to increased variability in red blood cell size and ultimately resulting in an elevated RDW-CV value (18). Third, similar to other malignant tumors, neuroblastic tumor cells can release significant amounts of reactive oxygen species (ROS), triggering oxidative stress responses that damage red blood cell membrane structures and disrupt intracellular metabolic processes, leading to increased heterogeneity in red blood cell size (31). Notably, RDW-CV holds independent significance in predicting tumor recurrence and patient mortality risk across various cancers, including colorectal cancer (32), breast cancer (33), diffuse large B-cell lymphoma (DLBCL) (34), and hepatocellular carcinoma (19). In these cancers, patients exhibiting higher RDW-CV values face an elevated risk of adverse outcomes, such as tumor recurrence or death, thereby underscoring its potential value in tumor assessment. Moreover, these hematological insights converge with coagulation function indicators, as both systems are intricately linked through tumor-induced inflammatory and angiogenic pathways.

Coagulation function indicators are closely associated with tumor initiation, progression, and metastasis. Elevated Fib levels in patients with malignant tumors significantly influence tumor cell growth, proliferation, migration, and apoptosis (35–38). For instance, in gastric cancer, breast cancer, endometrial cancer, and prostate cancer, high Fib serves as an independent prognostic factor by promoting lymphatic metastasis and reducing patient survival rates (35–39). Consistent with these findings, our study reveals that each 1-unit increase in Fib (g/L) is associated with a 53.9% lower risk of classifying neuroblastic tumor patients into localized stages (INSS 1-2) compared to advanced stages (INSS 3-4). This association is supported by three plausible mechanisms: firstly, Fib can combine with vascular endothelial growth factor-A (VEGF-A) secreted by neuroblastic tumor cells to promote angiogenesis in tumor tissues, ultimately leading to tumor growth and metastasis (37, 40, 41); secondly, it binds to fibrin receptors on tumor cells, enhancing the adhesiveness of these cells to the vascular endothelium of target organs, further promoting tumor metastasis (39); and thirdly, it may protect circulating tumor cells from natural killer (NK) cell-mediated elimination, thereby enhancing their metastatic potential (10). Additionally, TT is a test used to evaluate the functions of the coagulation, anticoagulation and fibrinolytic systems, providing insights into the content and quality of fibrin in plasma. Numerous studies have demonstrated that TT levels are significantly elevated in patients with tumors who experience microvascular invasion or metastasis (8, 9). For example, gastric neuroendocrine tumors (G-NET) with lymph node metastasis (42) and hepatocellular carcinoma with microvascular invasion (9) exhibit higher TT values compared to their non-metastatic counterparts. Additionally, TT levels in patients with colorectal cancer are significantly higher than those in patients with colorectal adenoma (43). Clinically, a 1-unit increase in TT is associated with a 45.7% reduction in the likelihood of patients being classified into the ganglioneuroma group compared to those with advanced neuroblastic tumors. This finding aligns with previous research; however, the underlying mechanisms require further investigation.

## 5 Conclusion

The logistic regression model developed in this study exhibited commendable performance in staging neuroblastic tumors and differentiating between advanced-stage neuroblastic tumors and ganglioneuromas (GN). The AUC was 0.867 for distinguishing localized from advanced-stage neuroblastic tumors and 0.941 for differentiating advanced-stage neuroblastic tumors from GNs. By incorporating non-invasive indicators such as age, Hb, and RDW-CV, this model effectively assesses tumor progression and pathological type during initial clinical diagnosis, thereby reducing the frequency of invasive examinations. This diagnostic approach significantly enhances the accuracy and efficiency of clinical diagnosis, providing essential reference information for diagnosing related diseases and formulating treatment plans. However, this study has certain limitations: firstly, it is a single-center retrospective design with a sample predominantly comprising the pediatric population, which may introduce regional and selection biases. Secondly, the analysis did not incorporate genetic markers (e.g., N-MYC, ALK), which may act as confounding factors and limit the interpretation of tumor heterogeneity. Additionally, while the detection indicators possess clinical value, they require further validation in conjunction with imaging and molecular pathology. Given these limitations, it is recommended to conduct multicenter, large-sample studies, construct multidimensional prediction models incorporating genetic markers, and evaluate the predictive efficacy of these models for prognosis through long-term follow-up to enhance the clinical application of non-invasive indicators in the precise diagnosis of neuroblastoma.

## Data Availability

The original contributions presented in the study are included in the article/**Supplementary Material**. Further inquiries can be directed to the corresponding author.

## Supplementary material

Supplementary Table 1 Demographic Comparison of Neuroblastic Tumors and Ganglioneuroma in Pediatric Patients.

Supplementary Table 2 Collinearity Diagnostics of Variables Entered into Unordered Multinomial Logistic Regression (Significant in Univariate Analysis).

Supplementary Table 3 Collinearity Diagnostics of Variables Retained in Multinomial Logistic Regression (Backward Stepwise).
