# Supplementary material for: Predictive model based on blood cell analysis and coagulation function indicators for neuroblastic tumors staging diagnosis
Source: Front Oncol. 2025 Jul 25;15:1575863. doi: 10.3389/fonc.2025.1575863 (PMC12331486; doi:10.3389/fonc.2025.1575863)
Supplement: Supplementary Table 1 — Demographic Comparison of Neuroblastic Tumors and Ganglioneuroma in Pediatric Patients. [file Table1.docx]

| **Suppl. Table 1. Demographic Comparison of Neuroblastic Tumors and Ganglioneuroma in Pediatric Patients** | | | | |
| --- | --- | --- | --- | --- |
| Indicators | Neuroblastic tumors  (n=112) | Ganglioneuroma (n=25) | Z/x2 | *p* |
| Age (months) | 23.5 (12-46.75) | 72 (53-108)a | -6.336 | <0.01 |
| sex |  |  | 0.461 | 0.497 |
| male | 68(60.7%) | 17(68.0%) |  |  |
| female | 44(39.3%) | 8(31.0%) |  |  |
| Regions |  |  | 0.388 | 0.533 |
| rural | 57(50.9%) | 11(44.0%) |  |  |
| urban | 55(49.1%) | 14(56.0%) |  |  |

| **Suppl. Table 2. Collinearity Diagnostics of Variables Entered into Unordered Multinomial Logistic Regression (Significant in Univariate Analysis)** | | | | | | |
| --- | --- | --- | --- | --- | --- | --- |
| Variable | B | SE | Beta | Variance Inflation Factor (VIF) | Tolerance | 95% CI for B  (Lower Bound,  Upper Bound) |
| intercept | 2.03 | 1.18 |  |  |  | (-0.312, 4.364) |
| Age (months) | 0.01 | 0.00 | 0.50 | 1.932 | 0.518 | (0.006, 0.013) |
| TT(s) | -0.03 | 0.03 | -0.10 | 2.447 | 0.409 | (-0.09, 0.034) |
| Fib (g/L) | -0.05 | 0.06 | -0.11 | 3.121 | 0.32 | (-0.17, 0.061) |
| PT(s) | 0.04 | 0.05 | 0.08 | 1.97 | 0.508 | (-0.063, 0.152) |
| D-Dimer (g/L) | 0.00 | 0.01 | 0.03 | 1.527 | 0.655 | (-0.014, 0.02) |
| LYM# (×10^9^/L) | -0.02 | 0.04 | -0.05 | 2.632 | 0.38 | (-0.096, 0.063) |
| NEU# (×10^9^/L) | 0.06 | 0.03 | 0.27 | 4.079 | 0.245 | (-0.002, 0.123) |
| MON# (×10^7^/L) | -0.01 | 0.00 | -0.25 | 3.267 | 0.306 | (-0.011, 0) |
| EOS# (×10^9^/L) | -0.46 | 0.31 | -0.12 | 1.457 | 0.686 | (-1.067, 0.156) |
| Hb (g/L) | 0.00 | 0.00 | 0.07 | 3.086 | 0.324 | (-0.005, 0.01) |
| MCV (fl) | -0.01 | 0.01 | -0.14 | 1.442 | 0.693 | (-0.031, 0.003) |
| RDW-CV (%) | 0.06 | 0.03 | 0.17 | 2.131 | 0.469 | (-0.011, 0.122) |
| NLR | -0.06 | 0.05 | -0.15 | 3.143 | 0.318 | (-0.156, 0.037) |

| **Suppl. Table 3. Collinearity Diagnostics of Variables Retained in Multinomial Logistic Regression (Backward Stepwise)** | | | | | | |
| --- | --- | --- | --- | --- | --- | --- |
| Variable | B | SE | Beta | Variance Inflation Factor (VIF) | Tolerance | 95% CI for B (Lower Bound,  Upper Bound) |
| intercept | 1.253 | 0.963 |  |  |  | (-0.651, 3.158) |
| Age(months) | 0.012 | 0.002 | 0.601 | 1.463 | 0.683 | (0.008, 0.015) |
| TT(s) | -0.011 | 0.026 | -0.037 | 1.589 | 0.629 | (-0.061, 0.04) |
| Fib (g/L) | -0.019 | 0.047 | -0.039 | 1.922 | 0.52 | (-0.111, 0.074) |
| MON# (×10^7^/L) | -0.002 | 0.002 | -0.092 | 1.39 | 0.72 | (-0.006, 0.002) |
| Hb (g/L) | -0.002 | 0.003 | -0.053 | 2.199 | 0.455 | (-0.009, 0.005) |
| RDW-CV (%) | 0.054 | 0.033 | 0.163 | 1.988 | 0.503 | (-0.012, 0.12) |
